# Discrimination and Precision of Continuous Glucose Monitoring in Staging Children With Presymptomatic Type 1 Diabetes

**DOI:** 10.1210/clinem/dgae691

**Published:** 2024-10-16

**Authors:** Elisabeth Huber, Tarini Singh, Melanie Bunk, Mayscha Hebel, Kerstin Kick, Andreas Weiß, Mirjam Kohls, Melanie Köger, Maja Hergl, Jose Maria Zapardiel Gonzalo, Ezio Bonifacio, Anette-G Ziegler

**Affiliations:** Institute of Diabetes Research, Helmholtz Munich, German Research Center for Environmental Health, Munich 80939, Germany; Institute of Diabetes Research, Helmholtz Munich, German Research Center for Environmental Health, Munich 80939, Germany; Institute of Diabetes Research, Helmholtz Munich, German Research Center for Environmental Health, Munich 80939, Germany; Institute of Diabetes Research, Helmholtz Munich, German Research Center for Environmental Health, Munich 80939, Germany; Forschergruppe Diabetes at Klinikum rechts der Isar, School of Medicine, Technical University Munich, Munich 80939, Germany; Institute of Diabetes Research, Helmholtz Munich, German Research Center for Environmental Health, Munich 80939, Germany; Institute of Diabetes Research, Helmholtz Munich, German Research Center for Environmental Health, Munich 80939, Germany; Institute of Diabetes Research, Helmholtz Munich, German Research Center for Environmental Health, Munich 80939, Germany; Institute of Diabetes Research, Helmholtz Munich, German Research Center for Environmental Health, Munich 80939, Germany; Institute of Diabetes Research, Helmholtz Munich, German Research Center for Environmental Health, Munich 80939, Germany; Faculty of Medicine, Center for Regenerative Therapies Dresden, Technische Universität Dresden, Dresden 01307, Germany; Paul Langerhans Institute Dresden of the Helmholtz Munich at University Hospital Carl Gustav Carus and Faculty of Medicine, TU Dresden, Dresden 01307, Germany; Institute of Diabetes Research, Helmholtz Munich, German Research Center for Environmental Health, Munich 80939, Germany; Forschergruppe Diabetes at Klinikum rechts der Isar, School of Medicine, Technical University Munich, Munich 80939, Germany

**Keywords:** continuous glucose monitoring, glucose variability, early-stage diagnosis, type 1 diabetes, pediatrics, disease progression

## Abstract

**Context:**

Staging and monitoring of presymptomatic type 1 diabetes includes the assessment for dysglycemia.

**Objective:**

To assess the ability of continuous glucose monitoring (CGM) to differentiate between islet autoantibody-negative controls and early-stage type 1 diabetes and explore whether CGM classifiers predict progression to clinical diabetes.

**Research Design and Methods:**

Children and adolescents participating in public health screening for islet autoantibodies in Bavaria, Germany, were invited to undergo CGM with Dexcom G6. In total, 118 participated and valid data was obtained from 97 [57 female; median age 10 (range 3-17) years], including 46 with stage 1, 18 with stage 2, and 33 with no islet autoantibodies.

**Results:**

Mean glucose during CGM in islet autoantibody-negative controls was high (median, 115.3 mg/dL) and varied substantially (interquartile range, 106.8-124.4). Eleven (33%) of the controls had more than 10% of glucose values above 140 mg/dL (TA140). Using thresholds corresponding to 100% specificity in controls, differences between controls and stage 1 and stage 2 were obtained for glucose SD, TA140, TA160, and TA180. Elevations in any 2 of these parameters identified 12 (67%) with stage 2 and 9 (82%) of 11 participants who developed clinical diabetes within 1 year. However, there was marked variation within groups for all parameters and poor consistency observed in a second CGM performed in 18 participants.

**Conclusion:**

This study demonstrated the potential of integrating CGM into staging and monitoring of early-stage type 1 diabetes. However, substantial improvement in the precision of CGM is required for its application in routine monitoring practices.

The presence of multiple islet autoantibodies is widely acknowledged as a robust biomarker for the diagnosis of early-stage type 1 diabetes. This diagnosis is complemented by staging, which relies on regular oral glucose tolerance tests (OGTT) and glycated hemoglobin A1C (HbA1c) testing for the differentiation of stage 1 (normoglycemia), stage 2 (dysglycemia), and stage 3 (hyperglycemia) type 1 diabetes (1). Diagnosing stage 2 type 1 diabetes is particularly critical to identify individuals who would benefit from the Food and Drug Administration-approved disease-modifying therapy Teplizumab (2, 3). Current monitoring guidelines include the use of the OGTT (4). Furthermore, several studies have proposed scores that incorporate parameters from the OGTT for predicting the progression time to the clinical manifestation of type 1 diabetes (5-9). However, compliance and adherence to the 2-hour OGTT can be challenging (4), and additional or alternative monitoring tools that could replace, optimize, and refine current staging strategies are encouraged (4).

Continuous glucose monitoring (CGM) has the potential to detect early disruptions in glucose metabolism without the need for venous blood sampling (10). Some research groups have explored the value of CGM in early-stage type 1 diabetes (11-14). Previous studies from Colorado and TrialNet found that more than 10% or 5%, respectively, of time spent with glucose levels above (TA) 140 mg/dL (7.8 mmol/L) is associated with rapid progression to stage 3 type 1 diabetes (15, 16). Other analyses have indicated that the 2-hour OGTT can more reliably predict progression than CGM (17). Further investigation, particularly in individuals identified from general population screening, is needed in this area.

The objectives of our study were to assess the ability of CGM parameters to differentiate between islet autoantibody-negative controls, children with early-stage 1, and children with stage 2 type 1 diabetes, to explore whether CGM classifiers predict rapid progression to clinical diabetes and whether CGM could replace the OGTT in classifying children and adolescents with early-stage type 1 diabetes.

## Methods and Study Design

Children and adolescents participating in the CGM-study were enrolled from the Fr1da public health screening program for islet autoantibodies (18). Between February 2015 and September 2023, 190,662 children underwent screening for islet autoantibodies facilitated by primary care pediatricians during routine medical check-ups in Bavaria, Germany. Screening eligibility encompassed children without known diabetes aged 1.75 to 5.99 years (February 2015 to March 2019) or 1.75 to 10.99 years (since April 2019). Early-stage type 1 diabetes was diagnosed when 2 or more islet autoantibodies against insulin, glutamic acid decarboxylase-65, insulinoma-associated antigen, or zinc transporter 8 tested positive in both an initial capillary blood screening sample and a second venous blood confirmatory sample as previously described (19-23). Families of children diagnosed with early-stage type 1 diabetes were invited to participate in a follow-up monitoring program including OGTT and HbA1c testing at a clinical referral center (18). Children and adolescents with early-stage type 1 diabetes aged ≥2 years who received care at the Munich clinical referral center were asked to participate in the CGM study [Supplementary Fig. S1 (24)]. Additionally, children and adolescents participating in the Fr1da study who screened islet autoantibody negative were enrolled into the CGM study as a control group. For all enrolled participants, HbA1c and OGTT were assessed on the same day as placing the CGM gear. The CGM study was approved by the institutional review board at the Technical University of Munich. Written, informed consent was obtained from the children's parents or legal guardians.

### Definition of Type 1 Diabetes Stages

Metabolic staging into stage 1 (normoglycemia) or stage 2 (dysglycemia) type 1 diabetes was based on the OGTT and HbA1c levels, in accordance with consensus criteria from the Juvenile Diabetes Research Foundation, Endocrine Society, and American Diabetes Association (1). Stage 1 type 1 diabetes was defined as 2 or more islet autoantibodies accompanied by normoglycemia based on OGTT and HbA1c results [fasting plasma glucose <110 mg/dL (6.1 mmol/L), 2-hour plasma glucose <140 mg/dL (7.8 mmol/L), plasma glucose <200 mg/dL (11.1 mmol/L) at 30, 60, or 90 minutes, and HbA1c < 5.7% (39 mmol/mol)]. Stage 2 type 1 diabetes was defined as 2 or more islet autoantibodies accompanied by dysglycemia based on OGTT or HbA1c results [fasting plasma glucose of 110-125 mg/dL (6.1-6.9 mmol/L) and/or impaired 2-hour plasma glucose of 140-199 mg/dL (7.8-11.0 mmol/L), and/or plasma glucose ≥200 mg/dL (11.1 mmol/L) at 30, 60, or 90 minutes, and/or HbA1c 5.7-6.4% (39-47 mmol/mol)]. Clinical type 1 diabetes (stage 3) was defined based on American Diabetes Association criteria: fasting plasma glucose ≥126 mg/dL (7.0 mmol/L) or a 2-hour plasma glucose of ≥200 mg/dL (11.1 mmol/L) in an OGTT; or HbA_1c_ > 6.5% (48 mmol/mol); or in children with classic symptoms of hyperglycemia, a random plasma glucose of >200 mg/dL (11.1 mmol/L) in the absence of unequivocal hyperglycemia. The first 3 criteria required confirmation by repeat testing for a stage 3 diagnosis.

### CGM

Participants underwent a 10-day period of CGM using the Dexcom G6 in blinded mode. The sensor was mounted by trained members of the study staff and was placed on the back of the upper arm or the abdomen, as preferred by the participant. Participants were blinded to the real-time CGM readings. Four different lots of sensors were used throughout the project. A recurrent period of CGM wear was possible after approximately 6 months and concurrent with an OGTT. For participants with repeated measurements, the first CGM measurement period was included in the overall analysis.

### Statistical Analyses

The initial 12 hours from each CGM data set were excluded from the analysis, following previous recommendations (15, 16). In addition, data sets were assessed blinded to child antibody and stage status, and those with implausible CGM readings were removed as indicated in Supplementary Fig. S2A-C (24). This included removal of partial or full data from 2 control participants and 7 participants with early-stage type 1 diabetes. Only data sets with more than 96 remaining hours were included. Glucose variability was quantified using the parameters SD and coefficient of variation (CV). Other parameters examined were the mean glucose concentration; the median proportion of time spent with glucose levels above (TA) 140 mg/dL (7.8 mmol/L; TA140), above 160 mg/dL (8.9 mmol/L; TA160), above 180 mg/dL (10 mmol/L; TA180), and above 200 mg/dL (11.1 mmol/L; TA200); the median proportion of time spent with glucose levels below (TB) 100 mg/dL (5.6 mmol/L; TB100) and below 80 mg/dL (4.5 mmol/L; TB80); and the maximum glucose and the mean glucose concentration between 0300 hours and 0600 hours.

The study numbers were based on previous findings (11). However, an interim analysis of values indicated that these assumptions were not valid in our study. Therefore, an exploratory analysis was performed to assess the CGM parameters in the 3 comparison groups and progression to stage 3 type 1 diabetes. Statistical analyses were performed using R software, version 4.3.0 (25). Variables were compared between islet autoantibody-negative control participants and stage 1 and stage 2 type 1 diabetes using the Kruskal–Wallis and Mann–Whitney U tests. Categorical data were compared using chi-square or Fisher's exact tests. Receiver operating characteristic (ROC) analyses were conducted to assess the ability of parameters to distinguish control participants and those with stage 2 type 1 diabetes. Kaplan–Meier analyses were applied to estimate the cumulative risk of developing stage 3 type 1 diabetes with data censored according to the length of follow-up and the log-rank test used to compare probabilities of outcome in participants stratified for each variable [performed using R package survival, version 3.5-7 (26)]. A 2-tailed *P*-value <.05 was considered significant.

## Results

From August 2022 to November 2023, a cohort of 212 children was approached for enrollment in the CGM study and invited to undergo a 10-day period of CGM using the Dexcom G6 in blinded mode. Among those approached, 118 agreed to participate, including 38 islet autoantibody-negative youth (control participants) and 80 with multiple islet autoantibodies. The data sets of 21 participants (5 controls, 10 with stage 1 and 6 with stage 2 type 1 diabetes) were subsequently excluded due to either sensor failure to record data for ≥96 hours, the recording of implausible data, or because they had progressed to stage 3 type 1 diabetes [Supplementary Fig. S1 and 2A-2C (24)]. This left 97 participants (57 female; median age 10 years, range 3-17 years) with data for analysis [Supplementary Fig. S1 (24)], comprising 33 controls without islet autoantibodies and 64 with multiple islet autoantibodies. Based on results from HbA1c and OGTT conducted during the same visit as the CGM sensor application, 46 of the participants with islet autoantibodies were diagnosed with stage 1 and 18 with stage 2 type 1 diabetes. Among the 64 participants with islet autoantibodies and valid CGM readings, 18 underwent a second CGM period after a median follow-up of 5.9 months [interquartile range (IQR) 5.6-10.8].

### Islet Autoantibody-negative Control Group

**Figure 1.**
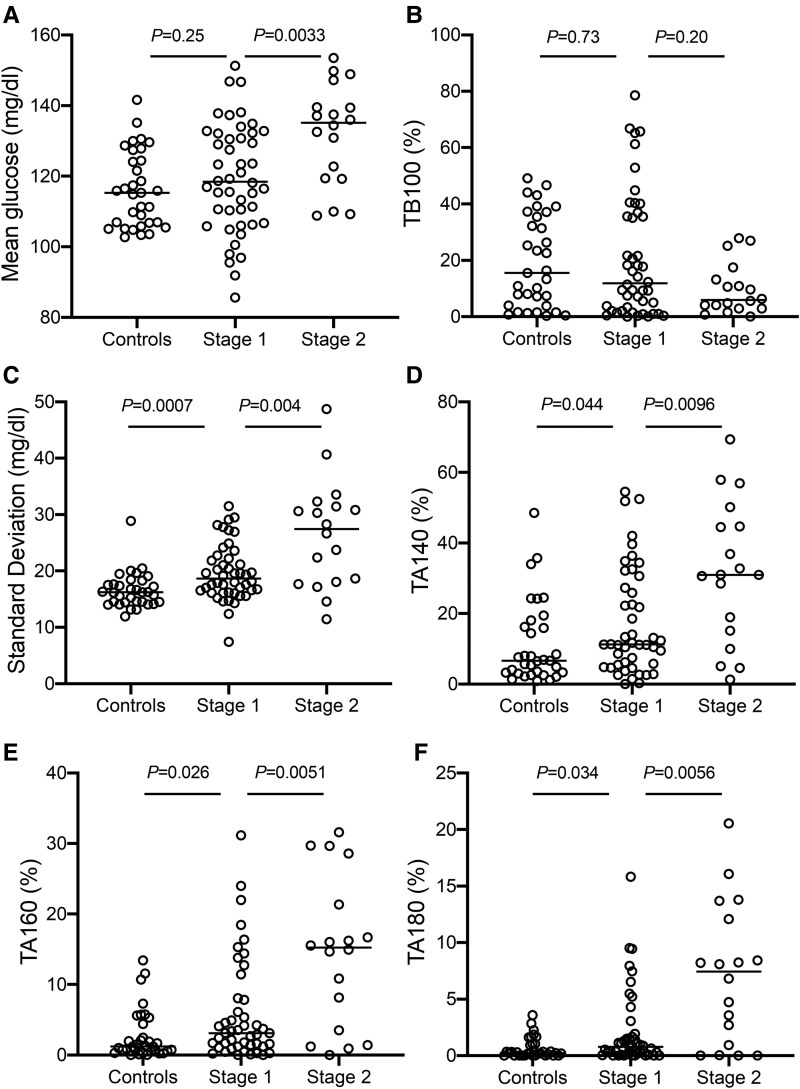
CGM metrics in islet autoantibody-negative youth and youth with stage 1 and stage 2 type 1 diabetes; (A) mean glucose (mg/dL), (B) SD (mg/dL), (C) CV (%), (D) TA140 (%), (E) TA160 (%), (F) TA180 (%). *P*-values indicate the level of significance between groups (Mann–Whitney U test). Abbreviations: CV, coefficient of variation; CGM, continuous glucose monitoring; TA, time above; TB, time below.

Overall glucose values as assessed by CGM in the 33 islet autoantibody-negative control participants ranged from a minimum 47 to a maximum 270 mg/dL (2.6 to 14.9 mmol/L). Mean glucose levels were unexpectedly elevated, with a median of 115.3 mg/dL (6.4 mmol/L) and a considerable glucose variability observed among control participants, ranging from 102.7 to 141.6 mg/dL (5.7 to 7.9 mmol/L) (Table 1, Fig. 1). High glucose levels and large variation were also observed for mean glucose between 0300 hours and 0600 hours [median, 106.3 mg/dL (5.9 mmol/L); range, 93.1-133.2 mg/dL (5.17-7.39 mmol/L)], a time expected to approach fasting levels. The TA140 and TA160 were 6.6% (IQR 3.2-16.2%) and 1.2% (IQR 0.6-4.4%), respectively, while the TB100 was only 15.5% (IQR 3.9-35.5%). Eleven (33%) of the 33 control participants had a TA140 that was greater than 10%, and 6 (18%) had a TA140 higher than 20%. A direct correlation between the mean glucose between 0300 hours and 0600 hours and the TA140 [r = 0.85; *P* < .0001; Supplementary Fig. S3A (24)] was observed. The correlation with mean glucose between 0300 hours and 0600 hours was less pronounced for SD (r = 0.35; *P* = .022) and was not observed for CV [r = 0.06; *P* = .76; Supplementary Fig. S3B, S3C (24)], suggesting that the large variation in mean glucose observed in control participants was primarily due to additive rather than multiplicative shifts in the glucose sensor values. The substantial variability observed at the individual level was evident in multiple sensor lots [Supplementary Fig. S4 (24)]. Overall, the findings in islet autoantibody-negative controls indicate that there is substantial variation in sensor values that is beyond the expectations of biological glucose variability.

**Table 1.** Participant characteristics and CGM values

CGM criteria	Control*^a^*	Stage 1	Stage 2	*P*-value		
	n = 33	n = 46	n = 18	Control vs	Control vs	Stage 1 vs
				stage 1	stage 2	stage 2
Age, years	11	9	9.5	.02	.18	.80
Median (IQR)	(10-13)	(7-11)	(7.3-11.8)			
Sex, male	11	20	9	.37	.25	.65
n (%)	(33.3)	(43.5)	(50)			
No. of days of data	9.4	8.8	9.3	.02	.53	.12
Median (IQR)	(9.2-9.4)	(7.9-9.4)	(8.8-9.4)			
Mean glucose mg/dL Median (IQR)	115.3 (106.8-124.4)	118.4 (107.6-131.8)	135.2 (120.2-139.5)	.25	<.0001	.003
SD glucose mg/dL Median (IQR)	16.2 (14.5-17.6)	18.7 (16.3-22.1)	27.5 (18.2-31.3)	.0007	<.0001	.004
CV, %	13.9	16.1	19.1	.002	.0003	.06
Median (IQR)	(13.1-15.4)	(13.9-18.2)	(15.6-23.7)			
Maximum glucose mg/dL Median (IQR)	199 (178-214)	212.5 (192.3-234)	268.5 (212.5-303.5)	.08	.001	.01
TB80, %	0.4	0.7	0.3	.29	.55	.13
Median (IQR)	(0.1-1.4)	(0.1-1.8)	(0-1.5)			
TB100, %	15.5	11.9	6	.73	.07	.19
Median (IQR)	(3.9-35.5)	(2.4-35.6)	(3.2-12.6)			
TA140, %	6.6	11.3	31	.04	.0003	.01
Median (IQR)	(3.2-16.2)	(5.5-26.9)	(16.1-44.6)			
TA160, %	1.2	3.1	15.2	.03	<.0001	.006
Median (IQR)	(0.6-4.4)	(1.3-7.4)	(4.7-20.2)			
TA180, %	0.2	0.8	7.4	.03	.0005	.006
Median (IQR)	(0-1)	(0.1-1.6)	(1.4-11.2)			
TA200, %	0	0.2	3.5	.06	.0003	.003
Median (IQR)	(0-0.2)	(0-0.5)	(2.5-4.9)			
Mean glucose 0300-0600 hours mg/dL Median (IQR)	106.3 (99.4-115.9)	111.2 (100.2-123.1)	119.6 (115.6-135.2)	0.26	<.0001	.005
>10% TA140	11	29	14	.009	.003	.27
n (%)	(33.3)	(63.04)	(77.8)			

Abbreviations: CGM, continuous glucose monitoring; IQR, interquartile range; TA, time above; TB, time below.

^*a*^Islet autoantibody-negative participants.

### Comparison of CGM Measurements Between Control Participants and Stage 1 or Stage 2 Type 1 Diabetes

Despite the unexpectedly high glucose levels and the high variability observed in control participants, there were significant differences between controls and stage 1 and stage 2 type 1 diabetes for several CGM parameters (Table 1, Fig. 1). Some CGM parameters were weakly associated with age [CV, r = −0.28; uncorrected *P* = .005; Supplementary Table (24)]. Differences both between controls and participants with stage 1 and between those with stage 1 and stage 2 type 1 diabetes were observed for the glucose SD, the TA140, the TA160, and the TA180 [Fig. 1, Supplementary Fig. S5 (24)]. These 4 discriminating parameters were utilized in subsequent analyses.

**Figure 2.**
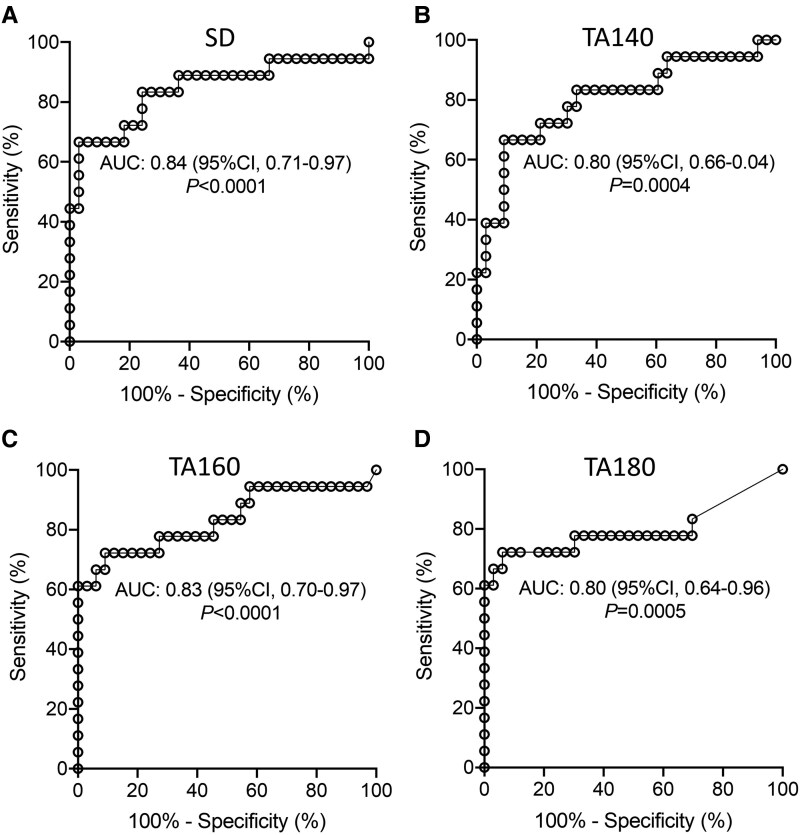
Receiver operator curve analysis between islet autoantibody-negative control youth and youth with stage 2 type 1 diabetes for (A) SD (mg/dL), (B) TA140 (%), (C) TA160 (%), and (D) TA180 (%). The area under the curve and *P*-values are indicated. Abbreviations: TA, time above; TB, time below.

ROC analysis was conducted for each of the discriminating parameters with the 33 islet autoantibody-negative participants assigned as controls and the 18 with stage 2 type 1 diabetes as cases (Fig. 2). The area under the curve (AUC) was 0.8 or higher for each of the 4 parameters. Thresholds corresponding to 100% specificity among the control participants were high [SD, 29 mg/mL (21.7 mmol/L); TA140, 50%; TA160, 14%; TA180, 4%] and well above previously suggested thresholds. Employing these thresholds identified 8 (44%) of the participants with stage 2 by the SD, 4 (22%) by the TA140, 11 (61%) by the TA160, and 11 (61%) by the TA180. Two or more of these 4 parameters above the threshold were observed in none of the controls, 6 (13%) with stage 1 (*P* = .032), and 12 (67%) with stage 2 type 1 diabetes (*P* < .0001). Therefore, although values and thresholds differed markedly from those suggested in previous studies, CGM was able to discriminate up to two-thirds of the participants with stage 2 type 1 diabetes from islet autoantibody-negative participants and show differences between stage 1 and stage 2 type 1 diabetes.

### CGM and Disease Progression

It was previously proposed that TA140 > 10% in children with islet autoantibodies is associated with a rapid progression to stage 3 type 1 diabetes (15). In our study, this threshold had a very low specificity and was associated with a low risk for stage 3 type 1 diabetes [1-year risk, 24%; 95% confidence interval (CI), 11-37; Supplementary Fig. S6 (24)], particularly in the those who had stage 1 type 1 diabetes (1-year risk, 6.9%; 95% CI, 0.1-15.9). Eight (44%) participants with stage 2 type 1 diabetes, and 3 (7%) of 46 with stage 1 type 1 diabetes developed clinical stage 3 type 1 diabetes within 1 year of their CGM test. Of these 11 participants, 5 (45%), 3 (27%), 7 (64%), and 7 (64%) were identified by the 100% specificity thresholds for SD, TA140, TA160, and TA180, respectively (Table 2). The corresponding positive predictive values (PPVs) were 38%, 57%, 41%, and 35%. Any 2 parameters above the 100% specificity thresholds identified 9 (82%) of those who progressed and had a PPV of 50%, which is similar to the stage 2 type 1 diabetes diagnosis in the participants.

**Table 2.** CGM vs OGTT for staging youth with multiple islet autoantibodies

CGM parameter	Stage 2 (n = 18)		Stage 1 (n = 46) (%)
	By OGTT (n = 9) (%)	By HbA1c only (n = 9) (%)	
SD >29 mg/dL*^a^*	6 (66.7)	2 (22.2)	3 (6.5)
TA140 > 50%*^a^*	1 (11.1)	3 (33.3)	3 (6.5)
TA160 > 14%*^a^*	7 (77.8)	3 (33.3)	7 (15.2)
TA180 > 4%*^a^*	7 (77.8)	4 (44.4)	9 (19.6)
Any 2 parameters	8 (88.9)	4 (44.4)	6 (13.0)
PPS ≥0.19	9 (100)	4 (44.4)	10 (21.7)

Abbreviations: CGM, continuous glucose monitoring; HbA1c, glycated hemoglobin A1c; OGTT, oral glucose tolerance test; PPS, Progression Prediction Score; TA, time above; TB, time below.

^*a*^Thresholds that correspond to 100% specificity in the islet autoantibody-negative controls.

**Figure 3.**
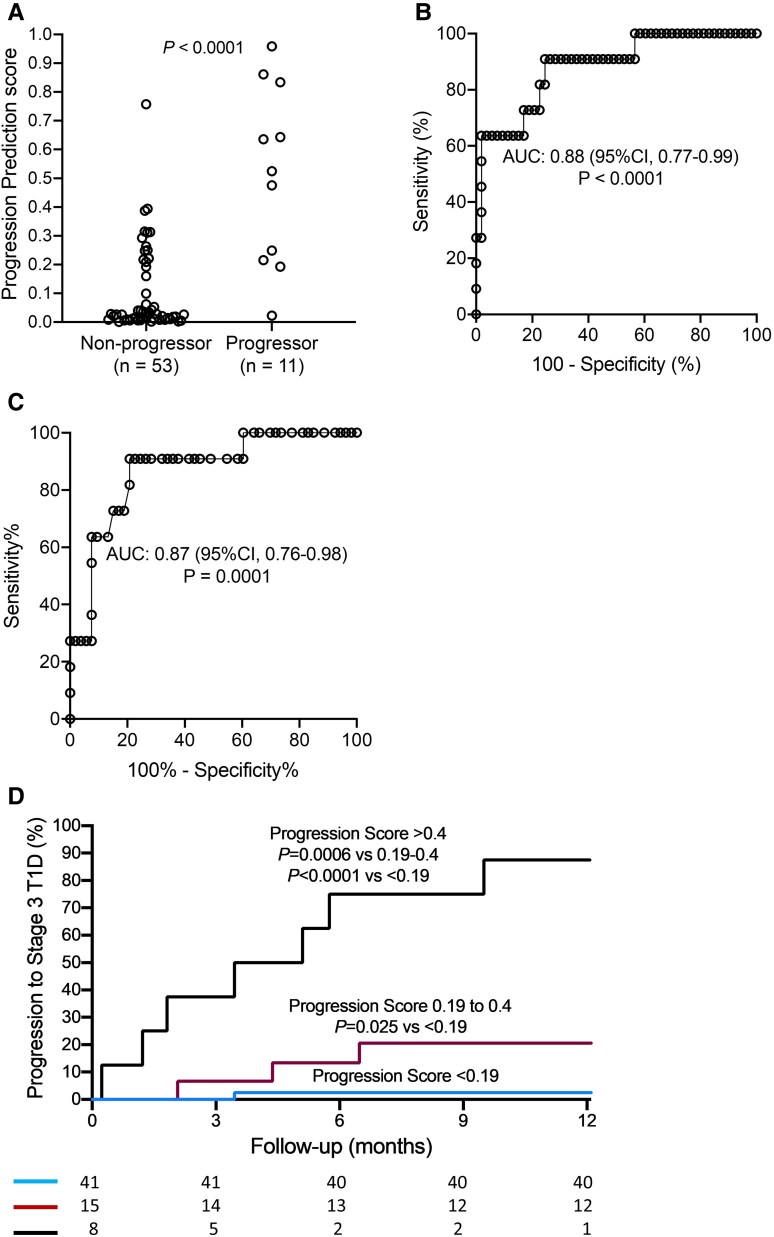
Logistic regression derived PPS values for progression to stage 3 type 1 diabetes. (A) PPS values in multiple islet autoantibody-positive youth who did and did not progress to stage 3 type 1 diabetes within 1 year. (B) ROC of PPS for discriminating multiple islet autoantibody-positive youth who did from those who did not progress to stage 3 type 1 diabetes within 1 year. (C) ROC of 120-minute glucose value in the OGTT for discriminating multiple islet autoantibody-positive youth who did from those who did not progress to stage 3 type 1 diabetes within 1 year. (D) Kaplan–Meier analysis of progression to stage 3 type 1 diabetes within 1 year by PPS. *P-*values for the Kaplan–Meier analysis were calculated using the log-rank test. Abbreviations: OGTT, oral glucose tolerance test; PPS, Progression Prediction Score; ROC, receiver operating characteristic.

A logistic regression analysis was performed to determine a composite progression score from the 4 parameters (Fig. 3A). The ROC curve showed discrimination (AUC, 0.88; 95% CI, 0.77-0.99; *P* < .0001), successfully identifying 10 out of the 11 who progressed (sensitivity, 91%), with a PPV of 42% at a threshold of 0.19 (Fig. 3B). In comparison, a single 120-minute glucose value of the OGTT had a ROC curve AUC of 0.87 [95% CI, 0.76-0.98; *P* = .0001; Fig. 3C (24)]. Progression to stage 3 type 1 diabetes within 1 year was 2.5% (95% CI, 0.1-7.5) for participants with scores <0.19, 20.6% (95% CI, 2.7-38.5) for participants with scores 0.19 to 0.4 (*P* = .025 vs <0.19), and 87.5% (95% CI, 67.5-99.5) for participants with scores >0.4 (*P* < .0001 vs <0.19 and *P* = .0006 vs 0.19 to 0.4; Fig. 3D).

These findings show the potential of CGM to predict a rapid progression to stage 3 type 1 diabetes. However, they also highlight the challenge of validating and applying previously defined values across studies.

### CGM as an Alternative to OGTT

The current criteria for defining stage 2 type 1 diabetes rely on an OGTT and the assessment of HbA1c. Although the OGTT is a well-established and standardized diagnostic tool, it poses certain challenges, particularly in young children. In the current cohort of 18 children or adolescents with stage 2 type 1 diabetes, 9 had stage 2 by OGTT criteria and 9 only by HbA1c values of 5.7% or higher (Table 2). Any 2 elevated parameters in the CGM identified 8 of the 9 with stage 2 by OGTT, a further 4 of the 9 with stage 2 by HbA1c only, and 6 (13%) of 46 participants with stage 1 type 1 diabetes. A progression score of 0.19 or higher identified all 9 of those with stage 2 by OGTT, a further 4 of 9 with stage 2 by HbA1c only, and 10 (21.7%) of the participants with stage 1 type 1 diabetes.

### Repeated Measurements

**Figure 4.**
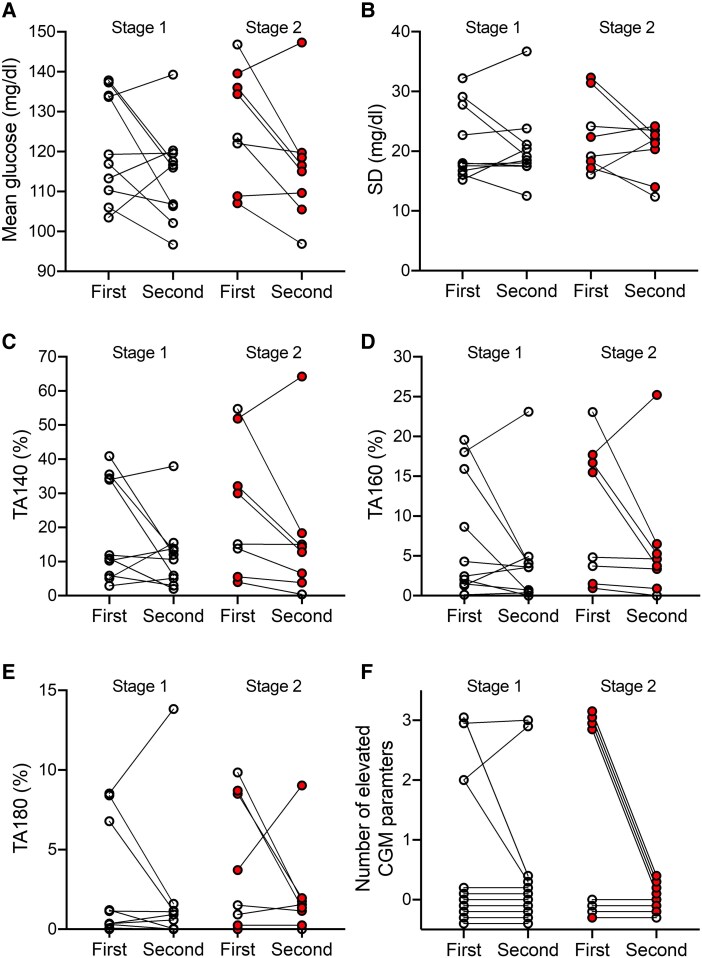
Comparison of CGM metrics in 18 participants with either stage 1 (left) or stage 2 (right) type 1 diabetes who had 2 periods of CGM assessments. (A) mean glucose (mg/dL), (B) SD (mg/dL), (C) TA140 (%), (D) TA160 (%), (E) TA180 (%), and (F) the number of parameters above 100% specificity values of islet autoantibody-negative controls. Individual participants are connected by lines. For participants with stage 2 type 1 diabetes, the stage 2 status is shown as a filled red circle for each of the 2 CGM assessments. Abbreviations: CGM, continuous glucose monitoring; TA, time above; TB, time below.

A second set of CGM measurements were performed in 18 of the participants with stage 1 or stage 2 type 1 diabetes (Fig. 4). In 9 of these 18, the mean glucose level in the second measurement differed by more than 10 mg/dL (0.6 mmol/L) compared to the first measurement. Specifically, 1 participant had an increase of 13 mg/dL (0.7 mmol/L), while 8 participants showed decreases ranging from 15 to 28 mg/dL (0.8-1.6 mmol/L) in the second measurement. The pronounced reductions were also observed for parameters such as TA140, TA160, and TA180.

Using the 100% specificity thresholds, there was substantial discordance in classifying participants as above and below thresholds for SD, TA140, TA160, and TA180. Similarly, of the 7 participants with 2 or more parameters above thresholds in their first measurement, 5, including 1 child who developed stage 3 type 1 diabetes, had values below thresholds for all parameters in their second measurement.

## Discussion

Utilizing CGM with Dexcom G6, our study delineated differences in CGM metrics between youth with stage 1 and stage 2 type 1 diabetes and islet autoantibody-negative controls. CGM parameters were also able to predict rapid progression to stage 3 type 1 diabetes in youth with multiple islet autoantibodies. However, variability between individuals, including islet autoantibody-negative controls, was high. Glucose values and CGM parameters obtained in controls often exceeded expected levels, resulting in low specificity for the previously proposed thresholds of 5% to 10% TA140. This underscores the potential of CGM for staging and monitoring early-stage type 1 diabetes while emphasizing the imperative for enhanced precision and standardization.

An unexpected finding was the high and varied values obtained in healthy islet autoantibody-negative controls. This variation was achieved across a period of 12 months and within multiple sensor lots, indicating that it was unlikely to be attributable to a low-quality sensor lot and could not be attributed to age. Furthermore, the reproducibility of repeat measurements was relatively poor. This substantial variation and higher than expected values hindered validation of previously identified thresholds for TA140. The TrialNet study group examined 7-day CGM in 105 relatives of individuals with type 1 diabetes with a median age of 16.8 years (16), and the Autoimmunity Screening for Kids study group investigated 7- to 10-day CGM in 91 participants with a median age of 11.5 years (15). Both studies identified TA140 as a suitable parameter to distinguish participants who had a high risk of progression to clinical diabetes, with TrialNet suggesting ≥5% and Autoimmunity Screening for Kids >10% as thresholds. Additionally, it was suggested that TA140 > 10% qualifies as part of the criteria for stage 2 dysglycemia (15). In our study, one-third of islet autoantibody-negative controls had a TA140 above 10% and only 7% of youth with stage 1 and TA140 > 10% progressed to stage 3 within 1 year. An important distinction from the earlier studies was that we used the Dexcom G6 sensor, which incorporates factory calibration, while the previous studies used Dexcom G4 sensors, which required calibration by the participants (15, 16). This may explain the higher mean glucose values observed in our study. The large interindividual variation within islet autoantibody-negative controls for measurements obtained between 0300 hours to 0600 hours supports this and suggests that measurements could be improved by including calibration and normalization of values.

Despite variation and imprecision, CGM effectively stratified islet autoantibody-negative controls, stage 1, and stage 2 type 1 diabetes. CGM was able to identify the majority of those with stage 2 without significant compromise in specificity. Elevated SD and TA140, TA160, and TA180 were discriminatory parameters. Two or more of these parameters and a score combining these 4 parameters were particularly effective. Differences between stage 1 and islet autoantibody-negative individuals were also observed in the TrialNet study (16), and increases in blood glucose concentrations have also been observed already around islet autoantibody seroconversion (27). Therefore, although stage 1 is defined as normoglycemia, there is impairment in some individuals in this stage. In line with this, we previously discerned a high-risk group within those with stage 1 by employing a score based on IA-2 antibody titer, HbA1c, and OGTT (5). The availability of practical and precise measures of beta cell function and glucose metabolism to include in such scores would strengthen their value and acceptance for monitoring of stage 1 type 1 diabetes and as surrogate endpoints in clinical trials performed at this early stage.

Our study had limitations. The relatively short follow-up period precluded insights into longer-term CGM metric predictions. Second, 46% of eligible individuals with early-stage type 1 diabetes declined participation, and from 16% of the enrolled individuals with early-stage type 1 diabetes no data was obtained or not included due to inadequate or implausible values. Therefore, both the acceptance and reliability of both blinded and unblinded CGM in early-stage type 1 diabetes need further assessment. The numbers of participants, especially those with stage 2 type 1 diabetes and those who progressed to stage 3 type 2 diabetes, were low, resulting in wide CIs for risk estimates. The thresholds and CGM parameters were highly influenced by the large variation in the islet autoantibody-negative controls. We have used these thresholds to demonstrate the potential of CGM metrics for staging and monitoring, but they are not intended to be a guide for implementation. In particular, the PPS is not validated and is provided as an example for how the CGM metrics may be elaborated in the future. Our results were based on the Dexcom G6 with sensors available in the period from August 2022 to November 2023. Future studies should consider comparisons across different CGM providers in early-stage type 1 diabetes. Participants in our study were relatively young and had a limited age range. The impact of age on CGM parameters in early-stage type 1 diabetes needs further assessment. The study was performed in a selected population of European descent living in and around Munich, Germany, and the findings may not be generalizable to other ethnic or racial groups.

In summary, this study demonstrated the potential of integrating CGM, either alone or in conjunction with the OGTT, in staging and monitoring early-stage type 1 diabetes. However, our study highlights the need for substantial improvement in the precision of CGM metrics before their broader application in routine monitoring practices.

## Data Availability

All datasets generated during and analyzed during the current study are not publicly available but are available from the corresponding author on reasonable request.

## Abbreviations

AUC: area under the curve
CGM: continuous glucose monitoring
CI: confidence interval
CV: coefficient of variation
HbA1c: glycated hemoglobin A1C
OGTT: oral glucose tolerance test
PPV: positive predictive value
ROC: receiver operating characteristic
TA: time above
TB: time below
